# Evaluation of the current status of Rehabilitation, Physical Medicine and Naturopathy education 10 years after the reform of the Medical Licensure Act – a nationwide survey of German Medical Universities

**DOI:** 10.3205/zma001080

**Published:** 2017-02-15

**Authors:** Beate Stock-Schröer, Roman Huber, Stefanie Joos, Petra Klose

**Affiliations:** 1 Karl and Veronica Carstens-Foundation, Essen, Germany; 2 University Medical Centre Freiburg, Centre for Complementary Medicine, Institute for Environmental Health Sciences and Infection Control, Freiburg, Germany; 3 University of Tuebingen, Medical Faculty, Tübingen, Germany; 4 University Hospital Tuebingen, Institute for General Practice, Tübingen, Germany; 5 University of Duisburg-Essen, Faculty of Medicine, Essen, Germany; 6 Kliniken Essen-Mitte, Department of Internal and Integrative Medicine, Essen, Germany

**Keywords:** Cross sectional area QB 12, Rehabilitation Medicine, Physical Medicine, Naturopathy, Evaluation, Survey, Teaching

## Abstract

**Introduction: **After the reform of the German Medical Licensure Act of 2003, Rehabilitation, Naturopathy and Physical Medicine were integrated into one discipline to be taught in Medical University. The aim of this survey is to determine the outcome of this change by evaluating the current status of education of these three disciplines based on the experience and satisfaction reported by lecturers responsible for teaching these subjects to medical students.

**Methods: **A questionnaire-based survey. A paper version of the questionnaire for each discipline was posted to each Medical University in Germany. The first part asked about the current status of teaching; the second part asked about facilities and requirements; the third part asked respondents to give information on their career and teaching experience in this subject

**Results: **The response rate was 51.5% for Rehabilitation, 48.5% for Physical Medicine and 60.6% for Naturopathy. A vast range of people and faculties were involved in the curricula. The percentage of each discipline taught was unevenly distributed: the major proportion being rehabilitation (38%), then naturopathy 34% lastly physical medicine with less than a third (28%). The main delivery of these disciplines was through lectures in plenary sessions. Modern teaching methods were not in evidence. Lecturers were generally pleased to be working with the combination of the three disciplines.

**Conclusion: **Future medical education should improve upon teaching coordination and aim towards a common curriculum for these three disciplines. Expected future changes to medical curricula will provide opportunities to improve the implementation of Rehabilitation, Physical Medicine and Naturopathy in teaching and research.

## Zusammenfassung

**Zielsetzung: **Seit der Änderung der Approbationsordnung für Ärzte im Jahr 2003 sind Rehabilitation, Physikalische Medizin und Naturheilverfahren verpflichtende Fächer für Medizinstudierende in Deutschland, die in einen Querschnittsbereich zusammengefasst sind (QB12). Ziel der Studie war es, die Organisation des QB12 und die Erfahrungen der Verantwortlichen aller drei Themenbereiche an den Fakultäten zu evaluieren.

**Methodik:** Es wurde ein Fragebogen erstellt und per Post an alle 37 medizinischen Fakultäten in Deutschland gesendet. Er sollte von allen Verantwortlichen für den QB12 ausgefüllt werden. Der erste Teil des Fragebogens enthielt Fragen zu Zuständigkeiten, Umfang und Format der Lehre, Organisation, Curriculum, Themenschwerpunkte und Zufriedenheit mit der Lehre. Im zweiten Teil der Umfrage wurde nach den Einschätzungen und Aussichten, der Ausstattung und dem Bedarf an Lehrmitteln und –personal gefragt. Am Schluss sollten die Verantwortlichen Informationen über ihre berufliche Situation, die Lehrerfahrung und Zusatzqualifikationen geben.

**Ergebnisse: **Die Rücklaufquote der Fragebogen betrug für Rehabilitation 51,5%, für Physikalische Medizin 48,5% und für Naturheilverfahren 60,6%. Sehr unterschiedliche universitäre Einrichtungen waren für die Lehre im QB12 verantwortlich. Das Vorhandensein von Curricula wurde für Rehabilitation in 38%, für Physikalische Medizin in 28% und Naturheilverfahren in 34 % angegeben. Die Art des Unterrichts reichte von Vorlesung bis Exkursion, problemorientiertes Lernen wurde nicht angeboten. Die Kosten für die Lehre wurden in 45% voll von den Universitäten übernommen. Die Lehrenden zeigten sich mehrheitlich zufrieden mit der Lehrsituation. Der Hauptbedarf wurde für mehr Unterstützung in der Organisation angegeben.

**Schlussfolgerung: **Die Heterogenität in der Lehre und der beteiligten Institutionen lässt vermuten, dass sich das Wissen der Medizinstudierenden in Rehabilitation, Physikalischer Medizin und Naturheilverfahren an den Universitäten in Deutschland erheblich unterscheidet. Die Koordination der Lehre sollte verbessert und Curricula vereinheitlicht werden.

## 1. Introduction

The German Approbation Regulation – the state license for doctors - was amended in 2003 and from that time Rehabilitation (Rehab), Physical Medicine (Phys. med.) and Naturopathy (NHV) were combined to form one course for medical students. This cross-sectoral unit -called QB12 in German- is the only obligatory teaching offer for these subjects for students during their medical studies in Germany. However, medical universities can offer elective courses whereby naturopathy, acupuncture and homeopathy are explicitly named disciplines [1]. 

In the years 2006 and 2009 an Association of Working Groups from German Medical Universities dealing with the research and teaching of naturopathy and complementary medicine (CAM) initiated a survey of all universities to evaluate QB12, focusing on NHV and CAM.

In addition, in 2009 a congress took place, in Bonn, which elaborated on several aspects of teaching NHV and CAM in medical studies in Germany [www.uniforum-naturheilkunde.de/seminarkongress/].

In 2004, the Society of Rehabilitation Sciences (DGRW) and the German Society of Physical Medicine published their recommendation for learning targets of all three disciplines to be grouped into one common curriculum [2]. A position paper, based on a survey of medical universities, was published five years later in 2009. This included ten recommendations on how to refine rehabilitation teaching and authors identified the need for, and proposed to have teaching coordinators; and the implementation of detailed curricula [3]. A reanalysis of the teaching situation in 2010, carried out by the Society of Rehabilitation Sciences (DGRW,) focused on new methods like E-learning and new examination and evaluation modalities in Rehabilitation [4]. 

The present piece of work evaluates all three disciplines together taught in the cross-sectoral QB12 unit at German Medical Universities. In addition to their experiences with QB12, lecturers were asked to give information on: course content; teaching methods and tutor requirements, wishes and criticisms.

## 2. Methods

In February 2014 questionnaires were sent by post to all 37 German Medical Universities. To ensure a high response rate, cover letters were addressed in person to the individual in charge of the course. If a named contact could not be detected by internet or telephone, offices of the Dean were asked to deliver the questionnaires.

Individuals in charge received three questionnaires and were asked to give one to each discipline. However, it was also permissible to fill in data for two or all three disciplines. A stamped, addressed envelope made it easier to return. The written surveys were completed anonymously.

At the beginning of April 2014 a written reminder was sent to all universities. Contacts who had confirmed their interest in participating were called and asked to fill in and return the completed questionnaires. 

All questionnaires returned by 31^st^ July 2014 were included in the analysis. The results presented in this paper are based exclusively on this data. 

The development of the questionnaire was based on preliminary surveys from 2006 and 2009, on our own professional experiences as well as a through a literature search. The proposed list of questions was presented and discussed at a working group meeting held in Berlin in January 2014. We invited all individuals responsible for dealing with the research and teaching of NHV and CAM from the working groups of all German Medical Universities. Moreover this questionnaire was sent to the German Society of Rehabilitation Sciences (DGRW) as well as to the German Society of Physical Medicine and Rehabilitation (DGPMR). They were asked to comment on the questionnaire or give suggestions of improvements and support the survey by sending the questionnaire to their contacts.

The survey was divided into three parts: Part One on the current status of teaching the QB12 unit. Part Two on lecturers’ needs and Part Three on lecturers’ careers and teaching experience.

In Part One: we asked for general statements on the organization, responsibilities, extent of teaching, main topics and finances. In Part two: we asked about facilities and requirements, learning materials or personal support. In Part three: we asked about sociodemographic data, qualifications and professional careers.

In addition, participants were asked how satisfied they are with the implementation of QB12 and the reasons given for a good or bad evaluation by the lecturers of the course.

## 3. Results

Four out of 37 universities refused to participate for various reasons: e.g. one university had no lectures in QB12 at this time. In total, 27 questionnaires were returned on time from the remaining 33 medical universities.

**Figure 1:**
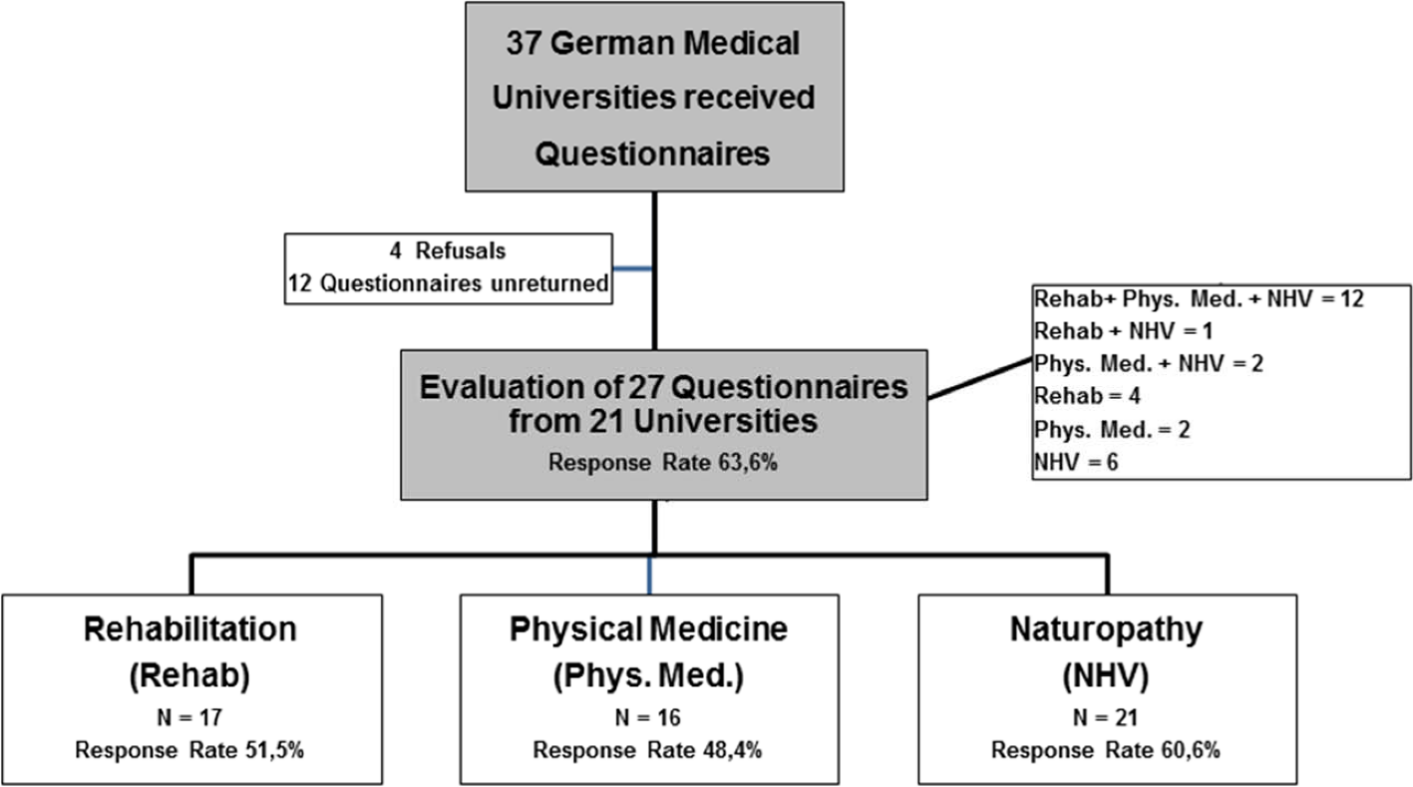
Survey Flowchart (measurement period 2/2014 to 8/2014)

Twelve questionnaires were filled in for all three disciplines, four for Rehab only, two for Phys. Med. and six for NHV only. One response for Rehab +NHV combined and two for Phys. Med. + NHV combined. Overall, 17 questionnaires were evaluated for the topic Rehab, 16 for Phys. Med. and 21 for NHV. Response rates for Rehab reached 51,5%, Phys. Med. 48,5% and NHV 60,6% (see figure 1 (Fig. 1)). 21 universities included QB12 in the winter and summer semesters, five universities only in the winter and one only during the summer semester.

### Personal Details and Qualifications

Men (N=17) filled in 63% of the answers and women (N=8) 30%. Two questionnaires did not include any personal details. Asked for their role in teaching QB12, 56 % of the participants (three women and twelve men) stated that they were responsible for the coordination of the teaching units, 37% (five women, five men) were commissioned to teach on site.

Out of the 27 questionnaires 19 answers were given in the section on personal additional qualifications. Among others the following qualifications were relevant to teaching Rehab, Phys. Med. or NHV: 1. specialist in physical and rehabilitative medicine, 2. additional titles of balneology and medical climatology, manual therapy, chirotherapy, naturopathy, physical therapy, physical therapy and balneology, rehabilitation.

#### Departments involved

Lecturers were asked to name the institution or department responsible for coordinating QB12 and which departments were involved in creating the syllabus. Less than half (N=10, 48%) of the identified 21 institutions responsible for teaching, named even one of the three disciplines explicitly in their title. Equally the words Rehab, Phys. Med and NHV did not appear in the names of the Departments identified as teaching this course in addition.

#### Payment of lectureships

Teaching of QB12 was an add-on, mainly unpaid or financed by external funding. NHV lecturers most often mentioned full financing of their teaching (see table 1 (Tab. 1)).

#### Curriculum and teaching units

In total, 13 (48%) questionnaires stated that a dedicated QB12 curriculum exists, of these: ten curricula cover all three disciplines; one covers Rehab only, one covers Phys. Med only, and one combines Rehab and NHV. Nine indicated that they have no curriculum, three stated they were working on a curriculum for all three disciplines and two didn´t answer this question. 

**Figure 2:**
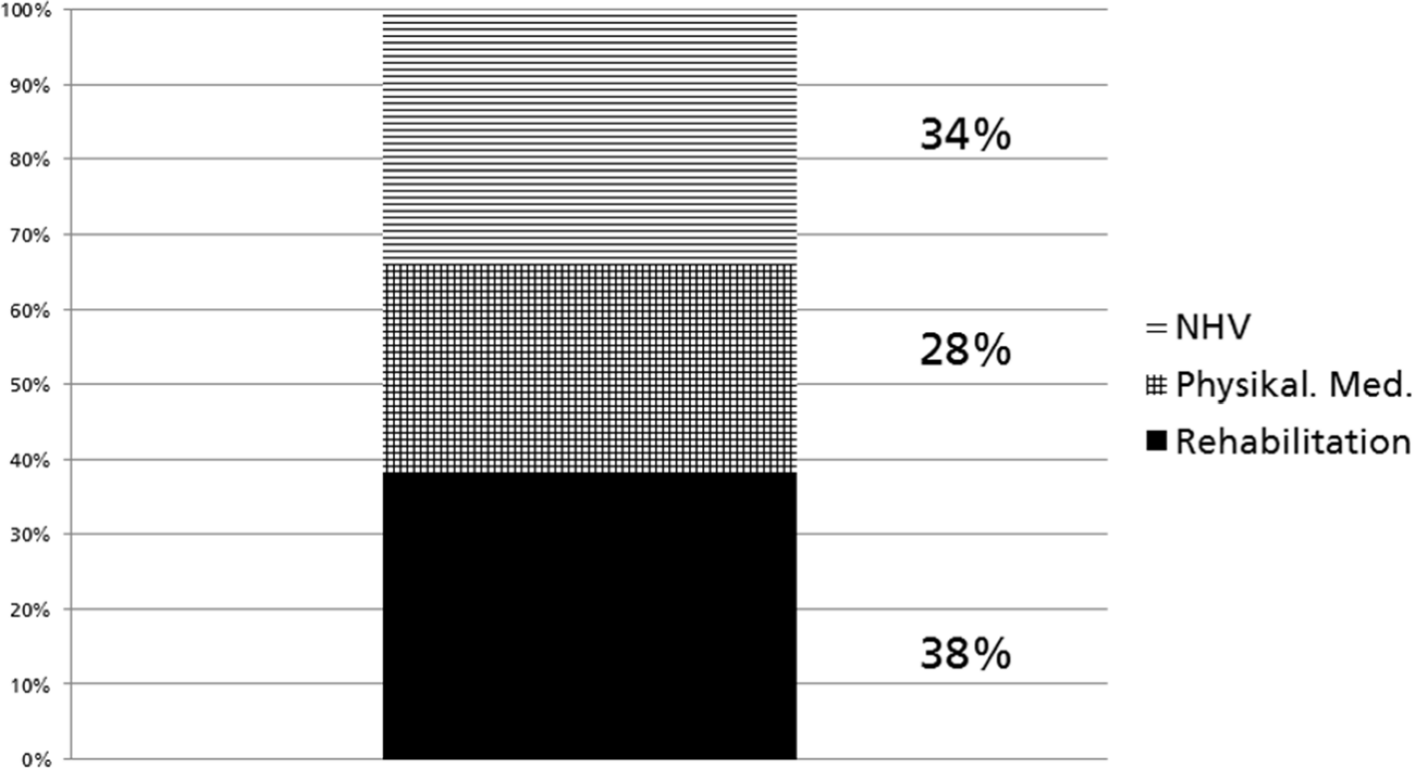
Distribuation of Teaching Units of all three QB12 Disciplines

Figure 2 (Fig. 2) shows the distribution of teaching units of all three QB12 disciplines, calculated for all participating universities.

#### Forms of Teaching and Contents

**Figure 3:**
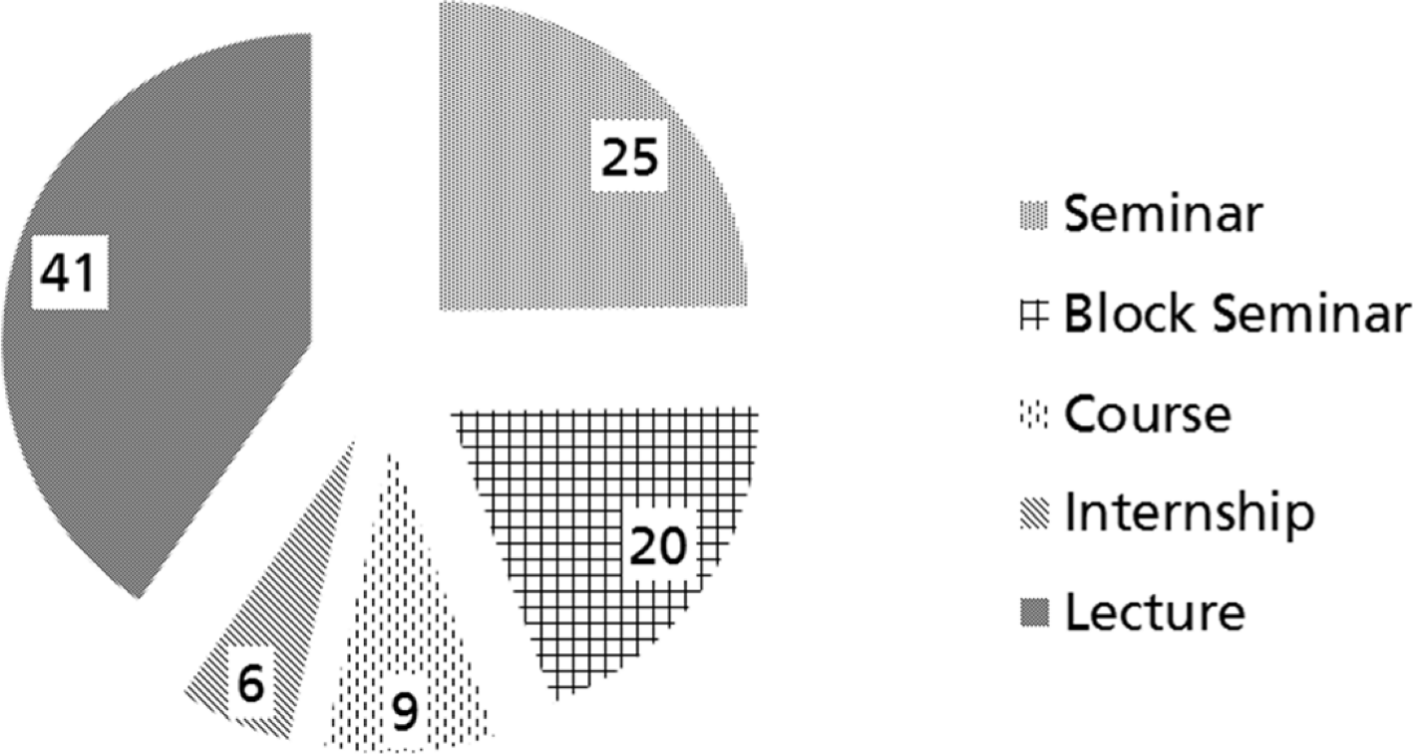
QB12 teaching methods (in %). Adapted from [19], with kind permission of S. Karger GmbH

Most teaching units (TU) were delivered through lectures and seminars. Problem – based – learning was not delivered in any of the participating universities. Three universities offered field visits e.g. to rehabilitation clinics. Figure 3 (Fig. 3) provides an overview of QB12 teaching methods, calculated for all participating universities.

Lecturers were asked to give a detailed description of every single discipline. In a free description field (see table 2 (Tab. 2)) they had the opportunity to describe extensively key topics of either Rehab, Phys. Med. or NHV. This showed that NHV lessons covered several other complementary and alternative therapies.

#### Positive and Negative Experiences 

Within the scope of free-text sections lecturers were asked to state their positive or negative experiences of teaching Rehab, Phys. Med. and NHV in QB12 (see table 3 (Tab. 3)).

#### Needs and Support 

In total, twenty (74%) of the participating lecturers had no need for additional teaching materials, two indicated that they needed slides or complete presentations to support their teaching of Rehab and Phys. Med.. Sixteen lecturers (59%) had no needs for further colleague support. Four had no need at all for external help to improve their lessons.

#### Students´ interest in these three disciplines

Finally, lecturers were asked to estimate to what extent student interest in QB12 has developed over the last years. More than one third (39%, N=10) out of all three disciplines state that interest in their discipline has stayed the same. Two lecturers in Rehab and NHV as well as three in Phys. Med. could not estimate student interest. Two Rehab lecturers, one lecturer for Phys. Med. and one for NHV stated a declining student interest. In contrast, five Rehab lecturers, six teaching Phys. Med. and eight responsible for NHV said that student interest in their disciplines had increased. 

## 4. Discussion

This is the first survey to examine lecture content, evaluate the relevant teachers and make a detailed comparison of the three QB12 subjects. In total QB12 information from 21 universities was evaluated, which corresponds to a response rate of 64% for 33 responses. Since the return rate is comparable for all three subject areas, statements can be made for each subject.

Almost half of the returned questionnaires were filled in for more than one topic, which suggests that teachers are often responsible for several QB12 subject areas.

The delivery of QB12 is heterogeneous for: time scale, weighting of the individual subareas, and for the responsibilities of the responsible individuals at each university. Heterogeneity has existed since the introduction of this cross-sectoral subject area [5]. The introduction of learning objective frameworks [3] and curricula does not seem to have brought about any changes. This is confirmed by the latest survey from 2015 [6]. Criticism has also been made of the widely differing content in other cross-disciplinary courses on offer at German Universities. In the section of their survey concerning the GTE (History, Theory and Ethics of Medicine), Möller et al. [7] concluded that the total number of hours varied according to the faculty. Differences also apply to teaching delivery, the existence of a curriculum and the teachers supporting the classes. For the cross-sectional area QB 13 (Palliative Medicine) Schiessl et al. [8] also revealed a great difference in the number of hours taught; and discussed whether this was only valid for QB13 curriculum or whether this was an expression of the design freedom of each individual faculty. The survey conducted by Plaumann et al. [9] also reflects teaching heterogeneity in the cross-disciplinary area of prevention and health promotion (QB10). This heterogeneity indicates that the outcome, namely the knowledge and abilities of the students taught at different universities, varies widely both qualitatively and quantitatively.

In Germany 60% of GPs delivering ambulatory clinical care, state that they use naturopathic or complementary medical procedures [10]. As the quota for research and teaching of QB12 subject areas in German universities is insufficient [11], future doctors are often unable to gain qualified knowledge and skills in these areas or can only acquire them on a point-by-point basis. This means that broader and advanced skills in the subjects can only to be learned outside the university at the relevant medical association and in the context of further education for physicians. It is assumed that the low level or missing structural incorporation, of Rehabilitation, Physical Medicine and Naturopathy at German Universities impairs research and teaching in these areas. This could lead to patients not receiving effective therapy options for Rehab, Phys Med and NHV. Moreover, it is understood that patients might utilize these therapy options without medical knowledge and might thereby endanger themselves, e.g. with interactions in the use of herbal medicines. For example, 40% of the patients diagnosed with ovarian carcinoma indicated that they received at least one additional herbal remedy without their physician's knowledge during chemotherapy, which in turn could limit the efficacy of chemotherapy [12].

Many overlaps exist in the teaching content of Rehabilitation, Physical Medicine and NHV. For example: Classical Naturopathy partially appears in Phys. Med. but aspects of it are taught again in a block-seminar for Naturopathy. If the universities set up a single unit to co-ordinate teaching in all three subjects, synergies could be created in favour of more content. The basis for better coordination could be a common curriculum, which so far only exists in a few universities. For example, in 2012 a qualitative survey was carried out: The eight heads of German University Institutions with a focus on NHV and Complementary Medicine believed that QB12 could only be effective if taught by teachers with a common vision of all of the three subject areas. Because of this common vision, it would then be possible to set and achieve objectives such as: networking in teaching and research; and the stabilization of research groups [13].

Additionally, the question arises of what core competencies universities should teach future physicians. In 2004, Mau et al. set up a learning objectives framework which lists teaching content for all three areas [2]. There is a common curriculum for naturopathy and complementary medicine, compiled across all universities [14]. These learning objective frameworks have not yet been centrally coordinated and are regarded as reference documents rather than obligatory ones. In the field of Rehabilitation Medicine, the curriculum has been coordinated by the Medical Association and has been recommended to the faculties. In the other two areas of Phys. Med and NHV no direction has been taken.

Practical and scientific independence should be strongly encouraged in medical students [15]. It is astonishing to compare the teaching methods collected by this survey with those in other fields of medicine and cross-disciplinary studies. QB12 lacks the introduction of those innovative and interactive teaching methods increasingly being used in other fields of medicine [16]. However, the therapeutic methods of Rehab, Phys. Med and NHV offer good opportunities for practical, innovative teaching. Modern practice-oriented teaching and testing procedures should increasingly be used, as proposed previously [5].

In this survey, lecturer’s commitment was the reason most frequently given for successful teaching. Good organization, a well rehearsed team and motivated students were also cited as important. In addition, the opportunities and conditions on site as well as the delivery tools seemed to be important factors for a good teaching course.

In 2010, Wiebelitz et al. concluded that the three subjects were a good choice of disciplines to teach jointly [17]. Despite heterogeneity, the differing proportions covered, and teaching responsibilities in the thematic areas, the present lecturers seem to be more satisfied than dissatisfied with the current situation. However, major changes in faculty design and teaching concepts are imminent. On the one hand, the model and reform study programs being permanently introduced do not explicitly provide cross-sectoral studies. There is a need to take a pre-emptive position on this. On the other hand, the entire medical studies program is on the move. Increasingly, traditional subject-specific teaching will give way to a competence-oriented transmission of content. Clinical and scientific content will be closely linked [18]. This represents an opportunity for QB12's popular and accepted therapies to be given a greater role in university teaching. 

## Competing interests

The authors declare that they have no competing interests.

## Figures and Tables

**Table 1:**
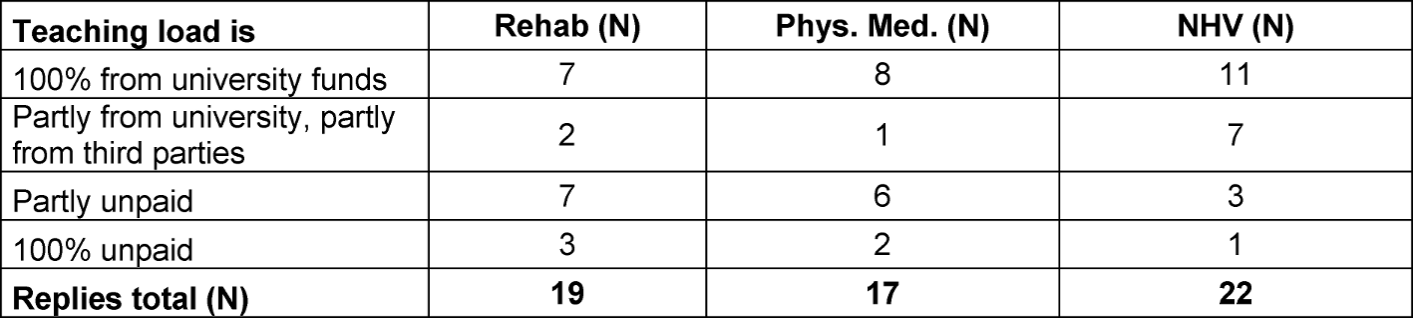
QB-12 payment modalities for teaching: rehabilitation, physical medicine and naturopathy (based on teachers’ estimates)

**Table 2:**
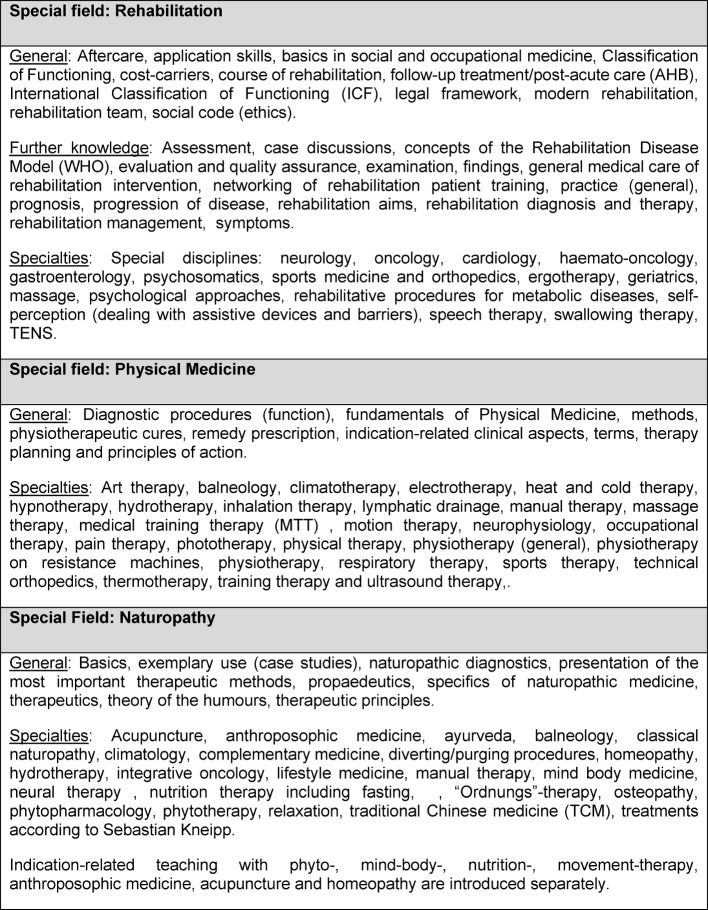
QB-12: Description of the three subjects and focus of teaching, ( from the general to the specific and alphabetically sorted). Adapetd from [19], with kind permission of S. Karger GmbH

**Table 3:**
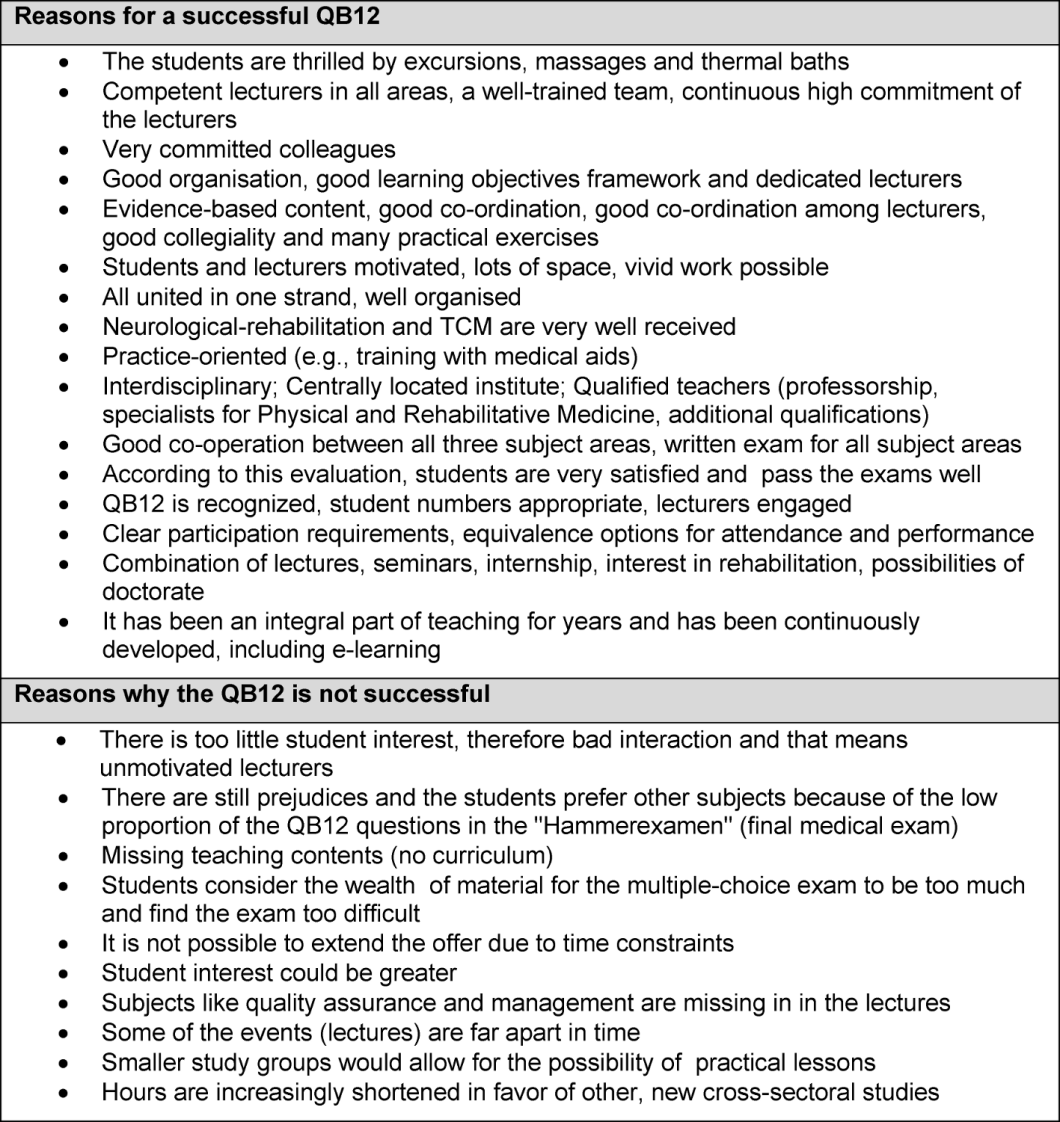
Respondents' free text replies to the question: Why, at their University, the implementation of QB-12 is doing well or badly
